# Characterisation, Sources and Flux of Unmelted Micrometeorites on Earth During the Last ~50,000 Years

**DOI:** 10.1038/s41598-018-27158-x

**Published:** 2018-06-11

**Authors:** M. Shyam Prasad, N. G. Rudraswami, Agnelo Alexandre de Araujo, V. D. Khedekar

**Affiliations:** Geological Oceanography Division CSIR-National Institute of Oceanography Dona Paula, Goa, 403004 India

## Abstract

Dust dominates extraterrestrial flux on the earth (30,000 tonnes/yr), however only ~5% of the cosmic dust survives atmospheric entry which is basically in two forms: melted and unmelted. Melted micrometeorites undergo transformational changes due to heating during atmospheric entry which obliterate evidences regarding their precursors. Unmelted micrometeorites (UMM) survive atmospheric entry with minimal alteration, they provide direct evidence for their parent bodies. Recent investigations unravelled a wide range of UMM, there are however no quantitative estimates of sources that contribute to the cosmic dust accreted by the Earth.

## Introduction

We present an unmelted micrometeorite collection that has so far the widest known range, comprising of a complex association of fine-grained scoriaceous particles, chondrules, composite particles, glassy meteorite matrices, individual mineral grains, refractory minerals and metal micrometeorites. Some types of particles are hitherto unreported. Also, for the first time quantitative estimations of micrometeorite flux from various meteorite groups/sub-groups are presented, which indicate a dominant component (80%) from carbonaceous chondrites, 15% from ordinary chondrites.

This investigation reveals micrometeorites are fragmented components of known groups of meteorites, confirming a strong link between micrometeorites and meteorites. The particles appear to be derived dominantly from asteroidal sources rather than cometary precursors.

Brownlee *et al*.^1^ were the first to identify two types of unmelted particles: (1) Fine-grained particles (fgMMs) (2) Coarse-grained assemblages of forsterites and enstatites (cgMMs) both had magnetite rims. They were assigned CI or CM carbonaceous chondrite origins. Subsequent investigations confirmed these broad types and their suggested origins^2,3^. UMM from the polar regions enabled the identification of particles from different sub-groups of carbonaceous chondrites^4,5^, ordinary chondrites^2,6–8^, and also from basaltic achondrites^9^. These investigations provided the primary link between meteorites and micrometeorites.

Melted micrometeorites from ten grab samples isolated from 293 kg of deepsea sediment were presented in our earlier investigation^10^. The magnetic fractions from five of these sediment samples were examined that led to the discovery of 195 UMM.

## Methods

During our earlier investigation^4^, we sieved 293 kg of deepsea surficial sediments of the Indian Ocean from ten closeby locations. We isolated 481 cosmic spherules from these samples^4^. These sediments covered a volume of 50 × 50 × 15 cm of the deepsea floor and based on the rates of sedimentation in this area, they were assigned a terrestrial age of 0 - ~50,000 years. In the present investigation, the magnetic fractions from five of those samples (Table 1), from which the spherules have been removed, are examined for unmelted cosmic particles. These samples cover a volume of 250 × 250 × 15 cm of the seafloor and by virtue of the earlier assigned dates^4^, they all have a terrestrial age range of 0 – ~50,000 years. The methods of sampling, magnetic separation of the particles, isolation of cosmic spherules and their results are described in (ref.^4^). In the present investigation, approximately 15000 magnetic particles have been mounted in 90 epoxy mounts, polished, observed and analysed using the CAMECA SX-Five EPMA at the CSIR-National Institute of Oceanography, Goa, India. Silicate and oxide standards were used for most of the samples, and for the metallic particles Fe-Ni or Fe-Ni-S standards were used. The cosmic particles were identified based on their textures, and chemical compositions as established in previous investigations^3,10^. The analysis was carried out at an accelerating voltage of 15 kV at a beam current of 12 nA. The beam diameter varied with respect to the type of particle analysed. For the scoriaceous particles, a beam diameter of 10 µm was used and several such analyses were carried out on each particle. For analysing individual features such as mineral phases etc. a beam diameter of 2 µm was utilized. Data reduction and correction were done using PAP model^11^. In addition, a JEOL JSM-5800LV SEM with an OXFORD ISIS energy dispersive x-ray analyser were also used for obtaining high-resolution images and for analysing specific areas/phases which were too small for the EPMA beam.

**Table 1:** Overview of the unmelted micrometeorites extracted from five deepsea surficial sediment samples.

Sample No.	No. of particles observed	No. of cosmic particles	Scoria-ceous	Composite particles	Glassy Matrix	Chondrule	Single crystals	Metal	Metal-rich chondritic	Refractory phases	Cosmic spherules	TOTAL No. of UMM {excluding cosmic spherules}
AAS38–165	2898	53	30	2	5	6	1	1	1	1	10	43
AAS38–155	2134	55	35	5	4	16	2	—	—	3	5	50
AAS38–153	2822	28	13	3	1	8	1	—	—	—	5	23
AAS38–147	3565	57	36	5	2	14	3	—	3	2	3	54
AAS38–139	2504	36	16	2	0	6	3	2	—	—	11	25
**TOTAL**	13923	229	130	17	12	50	10	3	4	6	34	195

The particles occur in the size range 83 × 70 µm to 732 × 392 µm (average size 220 µm) (Supplementary Table 1). In addition, we also found 34 melted micrometeorites adding to the earlier reported 245 melted micrometeorites from these samples^10^. The ratio of melted: unmelted micrometeorites is 59:41, similar to those from the polar regions^4^.

**Figure 1:**
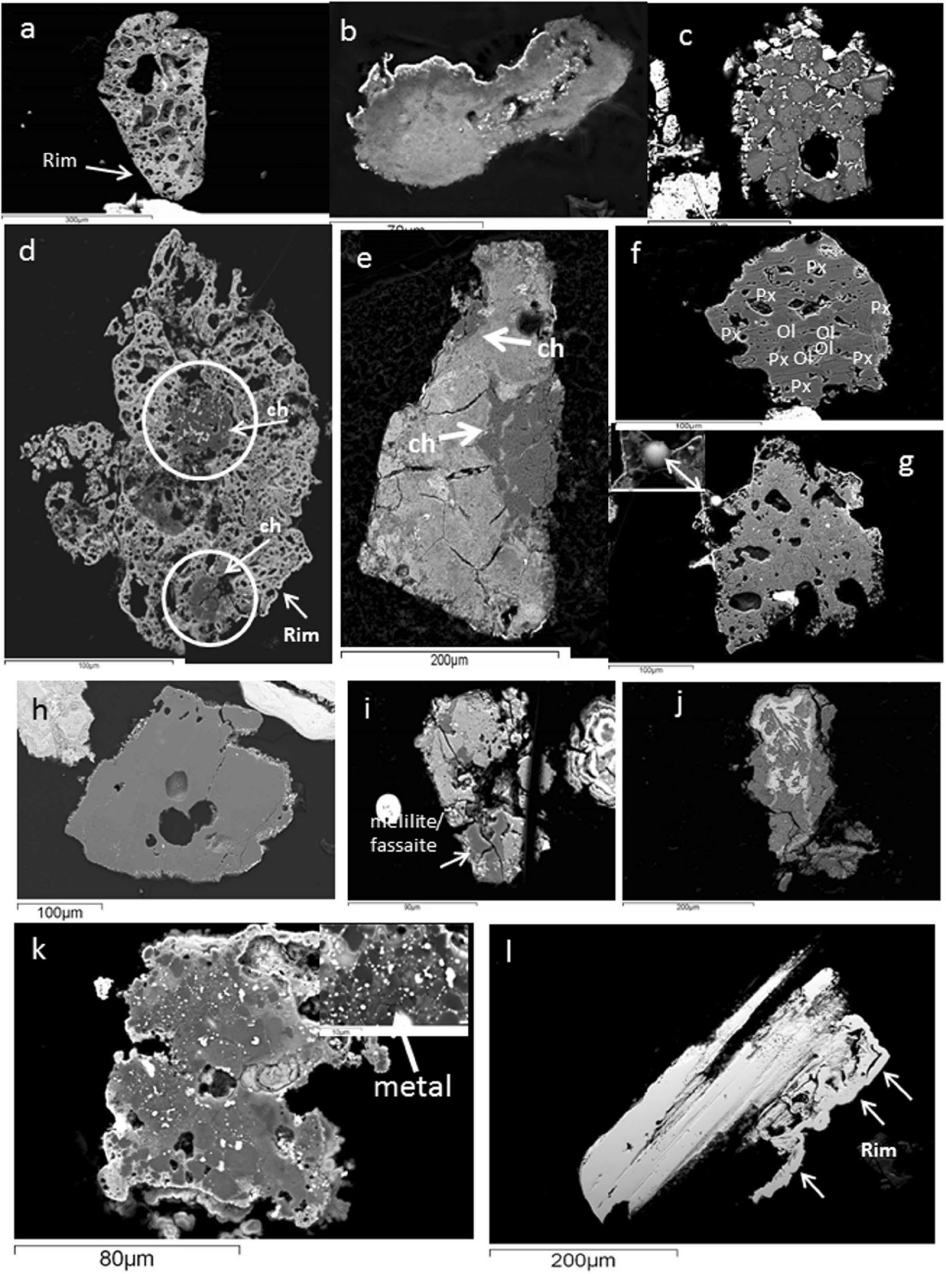
(**a**) AAS38-165 M-4 P-4. Typical scoriaceous (fgMM) particle. (**b**) AAS38-147 M-19 P-3. Highly fluffy scoriaceous particle with high volatile element contents. (**c**) AAS38-139 M-9 P3. Dusty olivine chondrule comprising entirely of reversely zoned olivine crystals. (**d**) AAS38-155 M-6 P-7. Composite particle (CM chondrite fragment) i.e., meteorite matrix with two small chondrules (marked ch). The top chondrule is olivine (Type IIA)and the bottom one (Type IIB) comprises of Mg-rich pyroxene. (**e**) AAS38-147 M-26 P-3. Composite particle (CM chondrite fragment) with two partial chondrules. The top semicircular portion contains olivine; the bottom larger chondrule is a POP chondrule with olivine surrounding Mg-rich pyroxene. (**f**) AAS38-155 M-7 P-2. cgMM a POP chondrule (Type IAB; CV chondritic) fragment comprising of olivine (ol) and pyroxene (px). (**g**) AAS38-165 M-2 P-4. Glassy, matrix that comprises of olivine normative composition with interstitial feldspar. Inset shows a small spherule development at the edge of the particle due to heating during entry. (**h**) AAS38-147 M-17 P-3. Individual mineral grain, Mg-rich enstatite with an oxidized rim. (**i**) AAS38-165 M-4P-2. Refractory inclusion of melilite/fassaite composition in a glassy matrix. (**j**) AAS38-139 M-15 P-1. Taenite crystal showing high levels of aqueous alteration that took place during its residence on the seafloor. (**k**) AAS38-147 M-18 P-5. Metal-rich chondriic particle. Closely-spaced and numerous blobs of kamacite composition. Inset a magnfied version of kamacite blobs. (**l**) AAS38-165 M-4 P-1. Native nickel with a prominent rim. The particle comprises of ~99% Ni. The rim contains very low levels of S and Fe (<1%).

**Figure 2:**
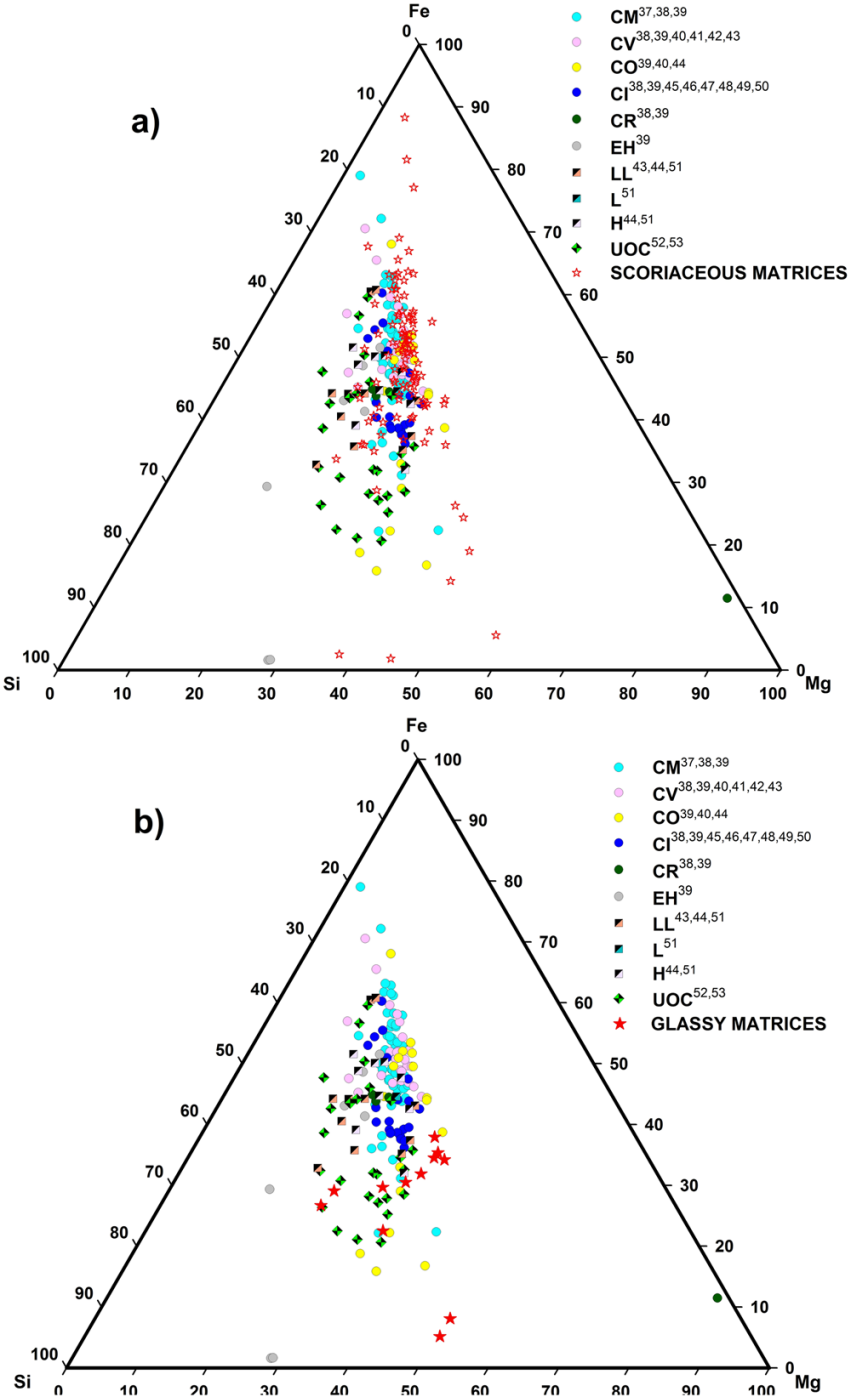
(**a**) Fe-Si-Mg plot showing compositions of scoriaceious particles (n = 107) in the present collection compared with the matrix compositions of different groups/sub-groups of chondritic meteorites. Data for different groups/sub-groups of chondritic meteorites are from: *CM chondrite data*^37–39^*; CV chondrite data*^38–43^; *CO chondrite data*^39,40,44^*; CI chondrite data*^38,39,45–50^*; CR chondrite data*^38,39^*; EH chondrite data*^39^*; LL chondrite data*^43,44,51^*; L chondrite data*^51^*; H chondrite data*^44,51^*; UOC chondrite data*^52,53^. (**b**) Triangular plot showing the compositions of glassy, hardened particles (n = 12) in comparison with the matrices of different groups/sub-groups of chondritic meteorites. Data for different groups/sub-groups of chondritic meteorites are from: *CM chondrite data*^37–39^*; CV chondrite data*^38–43^; *CO chondrite data*^39,40,44^*; CI chondrite data*^38,39,45–50^*; CR chondrite data*^38,39^*; EH chondrite data*^39^*; LL chondrite data*^43,44,51^*; L chondrite data*^51^*; H chondrite data*^44,51^*; UOC chondrite data*^52,53^.

Scoriaceous particles (fgMMs) form a major component of this collection (56%), occurring in a size range of 83–438 µm (average size 225 µm; SupplementaryTable 1). They are irregular, porous and encased in a thin magnetite shell (1–2 µm width) that formed due to partial melting during atmospheric entry (Fig. 1; Supplementary Fig. 1). Three particles (Fig. 1b) have fluffy, fine-grained textures with without mineral inclusions, enriched in sulphur (up to 2.56%). These particles could be the unaltered versions of the fgMMs, alternatively they could be more primitive, cometary particles in view of their highly porous nature, and high Fe-Ni-S contents^12^. In the Fe-Si-Mg triangular plot (Fig. 2a), a majority of the fgMMs trend in the compositions of CM chondrites, closely followed by CV, CO chondrites, and a few are CI chondritic. However, all these sub-groups show strong overlaps in the triangular plot^13^, therefore, their identification as carbonaceous chondritic based on their textures and chemical compositions can be assigned with greater confidence, whereas the sub-group assignments can only be tentative. The fgMMs are suggested to have been derived from the main belt asteroids as dust-sized debris^3^ showing affinities to the matrices of CM2, CR2 and CI carbonaceous chondrites^14^. It is also possible that some of the ultracarbonaceous fgMMs could be samples from hitherto unknown regions of the solar system^15,16^. Their survival in the unmelted/partially melted state suggests that they entered the atmosphere at low entry angles and velocities^17,18^ or they fragmented upon interaction with the atmosphere and have subsequently decelerated.

Fifty chondrules including fragments (29) are identified (Fig. 1c–f; Table 1, Supplementary Fig. 2), the fragments occur as cgMMs (Fig. 1f; Supplementary Fig. 3). Nineteen chondrules are enclosed in 17 composite particles i.e., scoriaceous matrix + chondrule (Fig. 1d,e; Supplementary Figs 2 and 3). A majority of these particles show affinity towards carbonaceous chondrites, therefore these particles provide substantive evidence connecting scoriaceous particles with the carbonaceous chondrites (Fig. 2).

Type II chondrules constitute 64% of the chondrule population, out of which, ~20% are IIAB (mostly POP chondrules) and 32% are Type IIB and 12% are Type IIA. All these chondrules are Fe-rich. Further, 30% of the chondrules belong to Type I, out of which 24% are of Type IB i.e., pyroxene chondrules. Four chondrules comprise entirely of dusty olivines (Fig. 1c) i.e., reversely zoned olivines with Ni-poor metal grains enclosed within a rim of Mg-rich olivine. Such chondrules have been observed mostly in UOCs and are suggested to have been produced by reduction of FeO-rich olivine^19^. In addition, one radiating pyroxene chondrule is found which generally occurs in ordinary chondrites^20^.

Based on their mineralogy (Olivine Fo-Fa contents^21^; Pyroxene En-Fs-Wo^22^) 68% of the chondrules appear to be sourced from carbonaceous chondrites (CC). Within the CC meteorites, CV (26%), CM 18%, CO (14%) and CR (10) are the main contributors. There is a large component of chondrules from ordinary chondrites (32%), with the following break up from different sub-groups: H:13%; LL:4%; L:7%, UOC8%. They can be identified to belong to different meteorite sub-groups only because of their unmelted nature. Chondrules (cgMMs) have been common among all UMM collections^7,8,14^.

Twelve hardened, glassy particles having Mg-rich olivine compositions (Fig. 1g; Supplementary Fig. 4) are observed. They do not have continuous magnetite rims indicating that these particles could be pieces of larger particles that fragmented during entry. Few particles contain glassy mesostasis having plagioclase composition ranging from bytownite to Na-rich feldspar. They cluster close together in the UOC part of the Fe-Mg-Si plot (Fig. 2b).

Four euhedral grains of pyroxene (Ca-poor enstatite) and three forsterites are found (Fig. 1h). They could have been dislodged from their parent sources and have entered the earth unmelted or were released from the parent meteorites during entry. The grains contain oxidized rims and have been observed in different collections^1,2,4^. Some of the enstatite grains could belong to CV chondrites, especially as CI and CM do not have such large enstatite grains.

Six particles enclose refractory inclusions, two of which are similar to melilite-rich CAIs observed in CV chondrites (Supplementary Fig. 5). Another particle containing melilite/fassaite inclusions shows similarity in composition to UOCs (Fig. 1i). Two fgMMs enclosing two spinels each, both having similar compositions are also observed (Supplementary Fig. 5d). All the four spinels in the present study have pure MgAl_2_O_4_ compositions that are typical of CAIs in CM and CV chondrites. One particle comprises entirely of diopside and enstatite and shows similarities to CAIs from CO chondrites^22^.

One native nickel particle (99% Ni; 386 × 185 µm size) is discovered, having a discontinuous melt rim of 8–10 µm thickness which also has pure nickel composition (Fig. 1I; Supplementary Fig. 6). The EPMA totals show consistently around 98–97%, with a mild presence of Fe (~0.1%). The rim, however, shows an average composition of: Ni = 98.4; Fe = 0.4; O = 0.7; and sulphur close to the detection limits. Due to heating during entry, the Fe and sulphur possibly migrated to the rim which explains the higher Fe contents of the rim. The particle remains otherwise unaffected by heat during entry which means that this particle experienced heat required for nickel to reach its melting point (1453 °C) and was cooled instantly. At high temperatures (>1000 °C), low oxygen partial pressures and minimal heating time (5–15 seconds) during atmospheric entry, the oxidation of nickel is severely curtailed^23^, it is 4 times higher at temperatures of ~600 °C^23^ however, by the time the particle reaches this temperature it would have solidified completely, therefore the particle remains largely unoxidized.

Terrestrial native nickel is uncommon and is reported to form due to hydrothermal alteration of ultramafic rocks^24^. Rare, native nickel is reported from metal cores of cosmic spherules^25^ and in meteorite fusion crusts^26^. In both these cases, the metal formed due to heating during atmospheric entry.

The native nickel particle here is geographically far removed from any hydrothermal source. Its irregular shape and the presence of a rim suggest that this particle entered the earth as unmelted, native metal and has not formed during entry. This is the first finding of pure nickel of extraterrestrial origin.

A sub-hedral taenite mineral with a magnetite rim is found showing aqueous alteration undergone during its residence on the oceanfloor (Fig. 1j). Similar particles have been presented by Prasad *et al*.^27^ and were suggested as possible precursors for the I-type spherules and further alteration would transform the particle into the serpentine mineral cronstedtite. The taenite can be a common occurrence in most chondritic meteorites or even in the iron and stony irons.

Three silicate particles contain unusual amounts of metal. Two of them contain metal (kamacite) in the reduced form within a silicate matrix (Fig. 1k; Supplementary Fig. 7), the other particle has oxidized metal (magnetite) along with a large forsterite grain. The particles also show primary aqueous alteration seen commonly in CI and CM chondrites. Although similar particles have been predicted to be the precursors for the G-type spherules^28^, metal-rich chondritic particles have not been reported so far.

**Figure 3:**
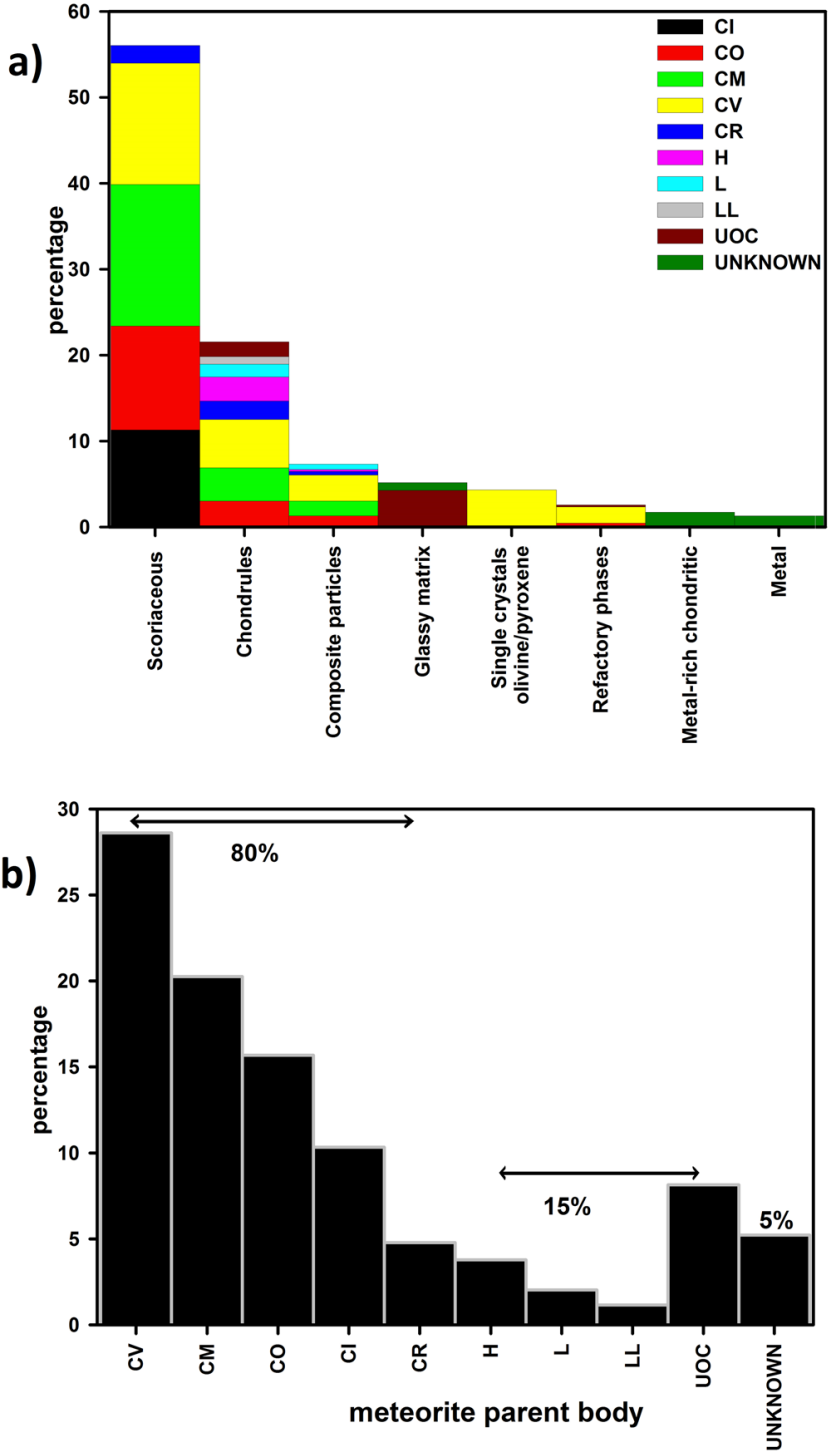
(**a**) Histogram showing different types of unmelted particles found in the present study and the percentages of particles sourced from different groups/sub-groups of chondritic meteorites in each category of UMM. (**b**) Break up of different types of parent bodies that constitute the present investigation of 195 unmelted micrometeorites.

In summary, based on their compositions and mineralogy the following parent meteorite groups/sub-groups are identified: 80% of the particles are fragments of components such as matrices, chondrules, refractory phases, that belong to carbonaceous chondrites (Fig. 3), out of which CV, CM, CO,CI and CR are the dominant sub-groups in this order (Fig. 3). The ordinary chondrites (H,L, LL) comprise 15% of the UMM. They occur mostly as fragments of chondrules, glassy matrices and few composite particles. The UOC shows a strong presence (8%) in the form of dusty olivine chondrules and glassy matrices. The achondrites which constitute a small percentage of all collections are not encountered in the present study – this could be a sampling artefact. Further, native nickel is a first time report and is not assigned any parent body because native nickel is not reported from any primary meteoritic source. In view of the above evidences these UMM provide an essential link between meteorites and micrometeorites. These results also have the potential to present the ground truth for astronomical observations.

Furthermore, Rudraswami *et al*.^29^ observed glass cosmic spherules that contain partly melted grains of pyroxenes. Glass cosmic spherules, in general, have pyroxene normative compositions. It is not kinetically possible for pyroxenes to crystallize during the minimal time available during atmospheric entry^30^, therefore the pyroxene crystals observed by are primary pyroxenes, in view of which such spherules could be Type 1B chondrules. This also substantiates the recent proposal by van Ginneken *et al*.^31^ that the parent bodies dictate the texture of cosmic spherules rather than the amount of heating experienced during atmospheric entry^4,6^. Therefore a substantial population of the melted micrometeorites also appear to be components of known meteorites.

Considering the large average particle size (220 µm), the size range (~90 to >700 µm), and their affinities to components from known meteorites, it appears that a majority of the particles have asteroidal origins. This is also supported by the distribution of the asteroidal dust sources, where the C-type asteroids that are a major dust source on the earth comprise >55% of the population whereas the S-type which are a source for ordinary chondrites are only 20%^32^. In addition, the more friable, hydrous asteroids break down during collisions and contribute dominantly to the dust in <300 µm sizes^33^. This collection contains a majority of scoriaceous particles (130/198), which are friable and contain high proportions of volatile elements. Their survival during entry demands conditions of extremely slow entry velocities and low entry angles, which for.asteroidal particles is estimated to be around 11 km/s^34^. Similar sized particles from the comets would have extremely high velocities in the vicinity of 70 km/s, therefore would not survive atmospheric entry. However, Nesvorny *et al*.^35^ suggested that ~85% of the dust-sized particles delivered to the Earth are sourced from the Jupiter family comets. Three fluffy, fine-grained scoriaceous particles having high volatile contents (Fig. 1) come close to the chondritic porous IDPs of cometary origin described in the literature and also found among Antarctic micrometeorites^15,16,35,5^. Cometary matter contains high percentages of Fe-Ni-S phases which are encased in a vesicular, silica-rich matrix^16^. If we consider such particles to be of cometary origin then a reasonable number of scoriaceous particles in the present collection can easily qualify to be from cometary sources.

The micrometeorite flux that impacts the top of the atmosphere is estimated at 30,000 tonnes/year^36^. Based on melted micrometeorite counts from the same samples we estimated a micrometeorite flux of 160 ± 70 tonnes/yr^10^. With the addition of 195 UMMs, using the same calculations the flux value for UMM is: 79 ± 35 tonnes/yr. Therefore the total estimated micrometeorite flux is 160 + 79 = 239 tonnes/yr. This is the time-averaged flux of particulate extraterrestrial matter that survives atmospheric entry both in the melted and unmelted forms in the deepsea calculated for the last ~50,000 years.

## Electronic supplementary material

Supplementary Figures

Supplementary Table 1
