# Supplementary material for: Characterisation, Sources and Flux of Unmelted Micrometeorites on Earth During the Last ~50,000 Years
Source: Sci Rep. 2018 Jun 11;8:8887. doi: 10.1038/s41598-018-27158-x (PMC5995856; doi:10.1038/s41598-018-27158-x)
Supplement: Supplementary file 1 — Supplementary Figures [file 41598_2018_27158_MOESM1_ESM.pdf]

SUPPLEMENTARY INFORMATION FOR

CHARACTERISATION, SOURCES AND FLUX OF UNMELTED MICROMETEORITES ON EARTH DURING THE LAST ~50,000 YEARS

M. Shyam Prasad*, N.G. Rudraswami, Agnelo Alexandre de Araujo, V.D. Khedekar

Geological Oceanography Division, CSIR-National Institute of Oceanography, Dona Paula, Panaji, Goa- 403004, India.
*Corresponding author. E-mail: shyam@nio.org

SUPPLEMENTARY FIGURES

SUPPLEMENTARY FIGURE 1: Scoriaceous micrometeorites
SUPPLEMENTARY FIGURE 2: Composite particles enclosing chondrules.
SUPPLEMENTARY FIGURE 3: Chondrule Fragments.
SUPPLEMENTARY FIGURE 4: Hard, glassy matrices
SUPPLEMENTARY FIGURE 5: Refractory phases.
SUPPLEMENTARY FIGURE 6: Native nickel particle.
SUPPLEMENTARY FIGURE 7: Metal-rich chondritic particles.

SUPPLEMENTARY FIGURE 1

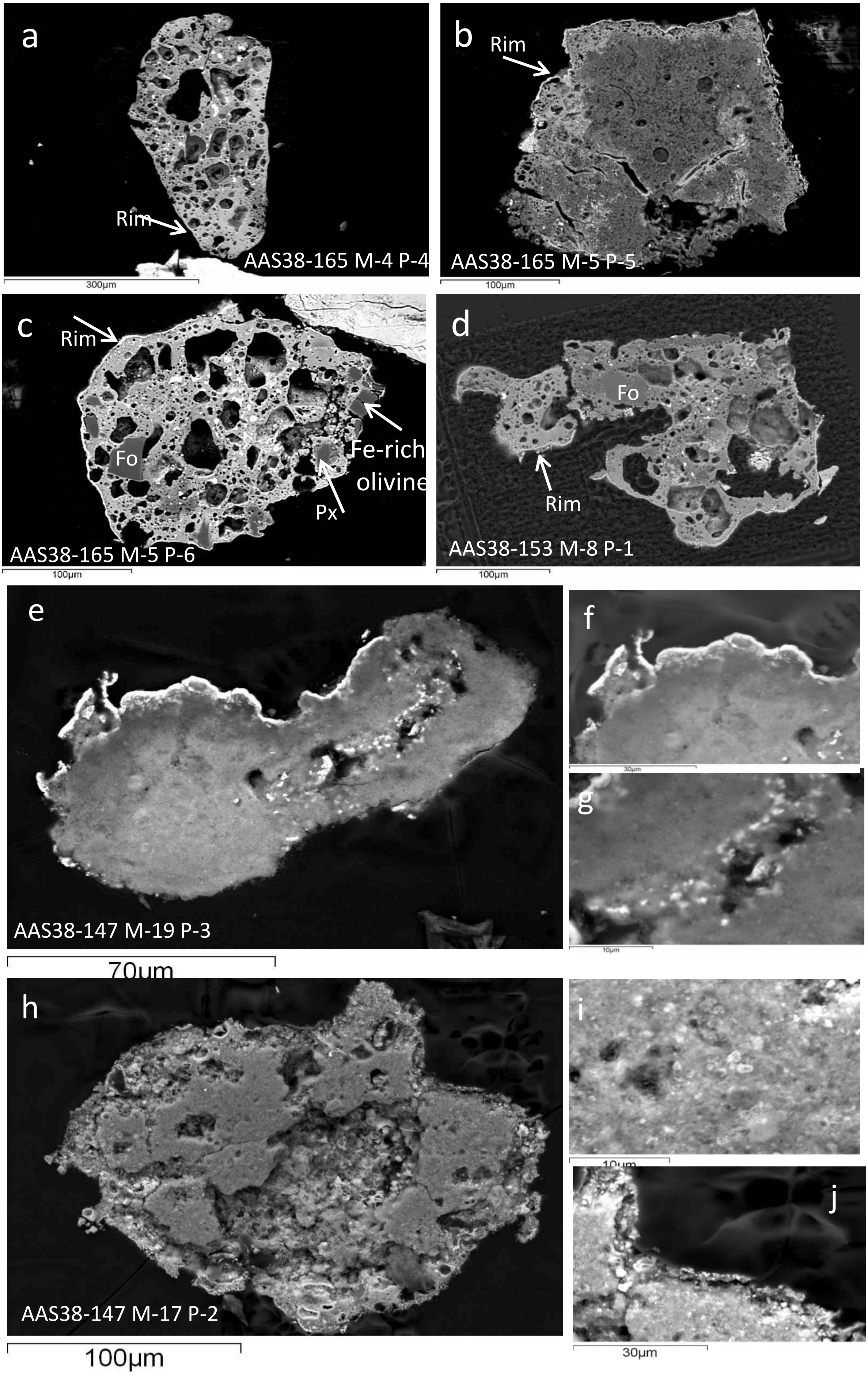

SUPPLEMENTARY FIGURE 1 : Scoriaceous micrometeorites.

All of them have a thin magnetite rim that surrounds them. The rim developed during atmospheric entry due to minimal heating and oxidation. The rim comprises of magnetite crystals. They are not part of a larger meteorite that fragmented during entry. They range from featureless (**a,b**) to those that contain forsterites (Fo), Fe-rich olivine grains (**c,d**) and also pyroxenes (Px) (**c**).

e-g and h-j. Two particles show very fluffy, fine-grained, featureless textures without any inclusions of any mineral grains. One of which (AAS38-147 M-19 P-3) shows a fairly prominent rim (**f**), and some magnetite formation due to heating during entry (**g**). The Other (AAS38-147 M-17 P-2) has experienced even lesser heating during entry. Both these particles show high enrichment in volatile elements such as sulfur and Na when compared with the other scoriaceous particles. It is highly porous (**i**) and has a discontinuous rim (**j**). It is possible, that some of the other scoriaceous particles which show somewhat coarser grained textures and numerous voids some of which are lines with metal/sulfur, may have been similar in texture. However, due to a more rigorous entry and having experienced higher amounts of heating during entry the coarser grained particles have had their volatile contents decreased. These have heated up, expanded in size and produced void spaces.

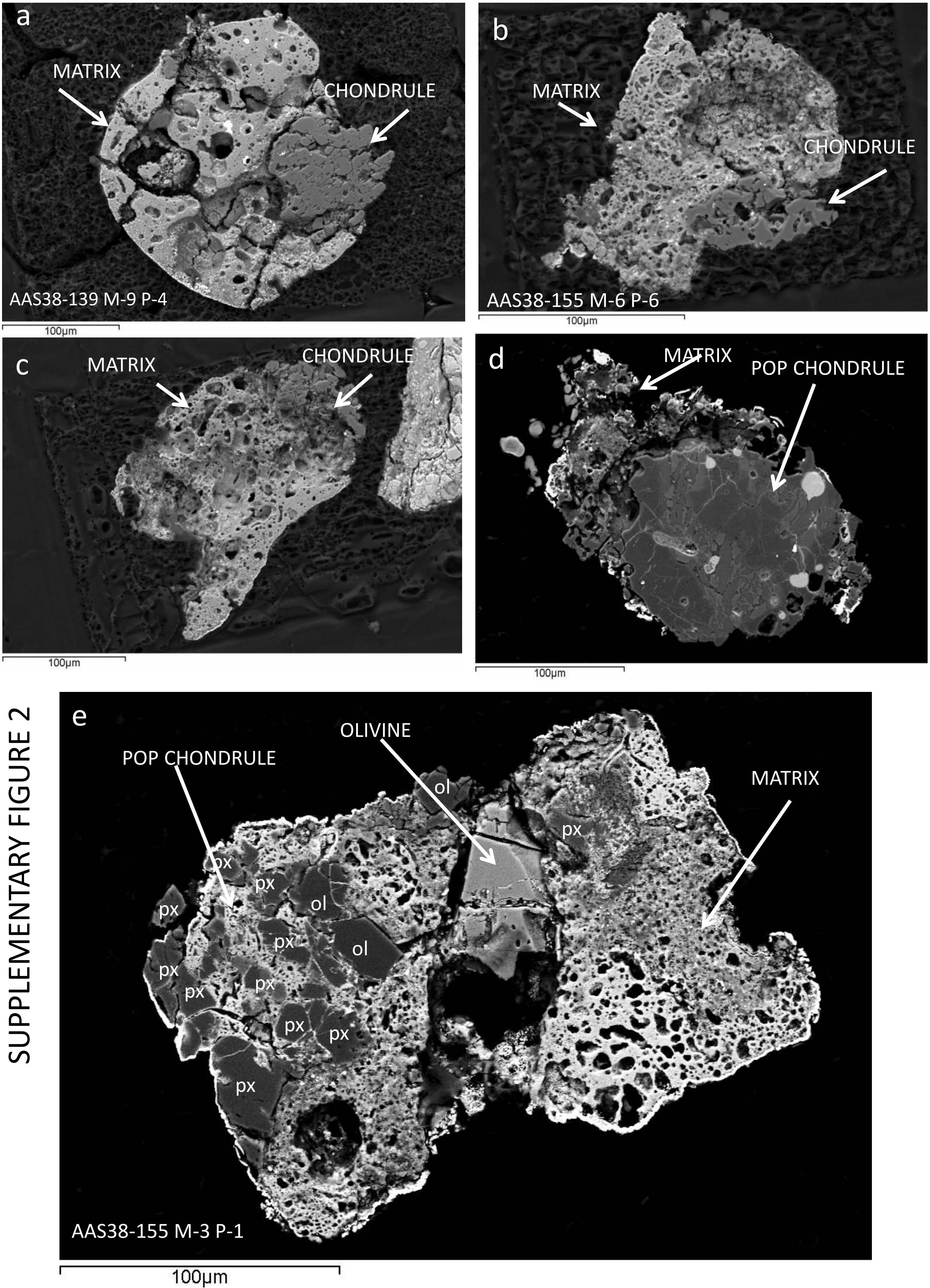

SUPPLEMENTARY FIGURE 2

SUPPLEMENTARY FIGURE 2 : **Composite particles enclosing chondrules.**

a. **AAS38-139 M-9 P-4**. Scoriaceous spherule enclosing a pyroxene chondrule. This particle has undergone partial heating during entry but still retains the identity of both the components i.e., matrix and chondrule.

b. **AAS38-155 M-6 P-6**. A scoriaceous particle with an enstatite chondrule (Type IB, CV chondritic). The chondrule has undergone some distortion due to heating during entry. A part of a discontinuous magnetite rim is visible in the top left area of the particle.

c. AAS38-155 M-4 P-2. A partial chondrule (Type IB; CV chondritic)enclosed within a scoriaceous particle.

d. AAS38-153 M-19 P-3. a POP chondrule (Type IIAB, CR chondritic) with oxidized metal blobs along with some matrix at the top left portion of the image.

e. AAS38-155 M-3 P-1. POP chondrule (Type IIAB; CO chondritic) enclosed in a scoriaceous matrix. A prominent rim surrounding the specimen is visible.

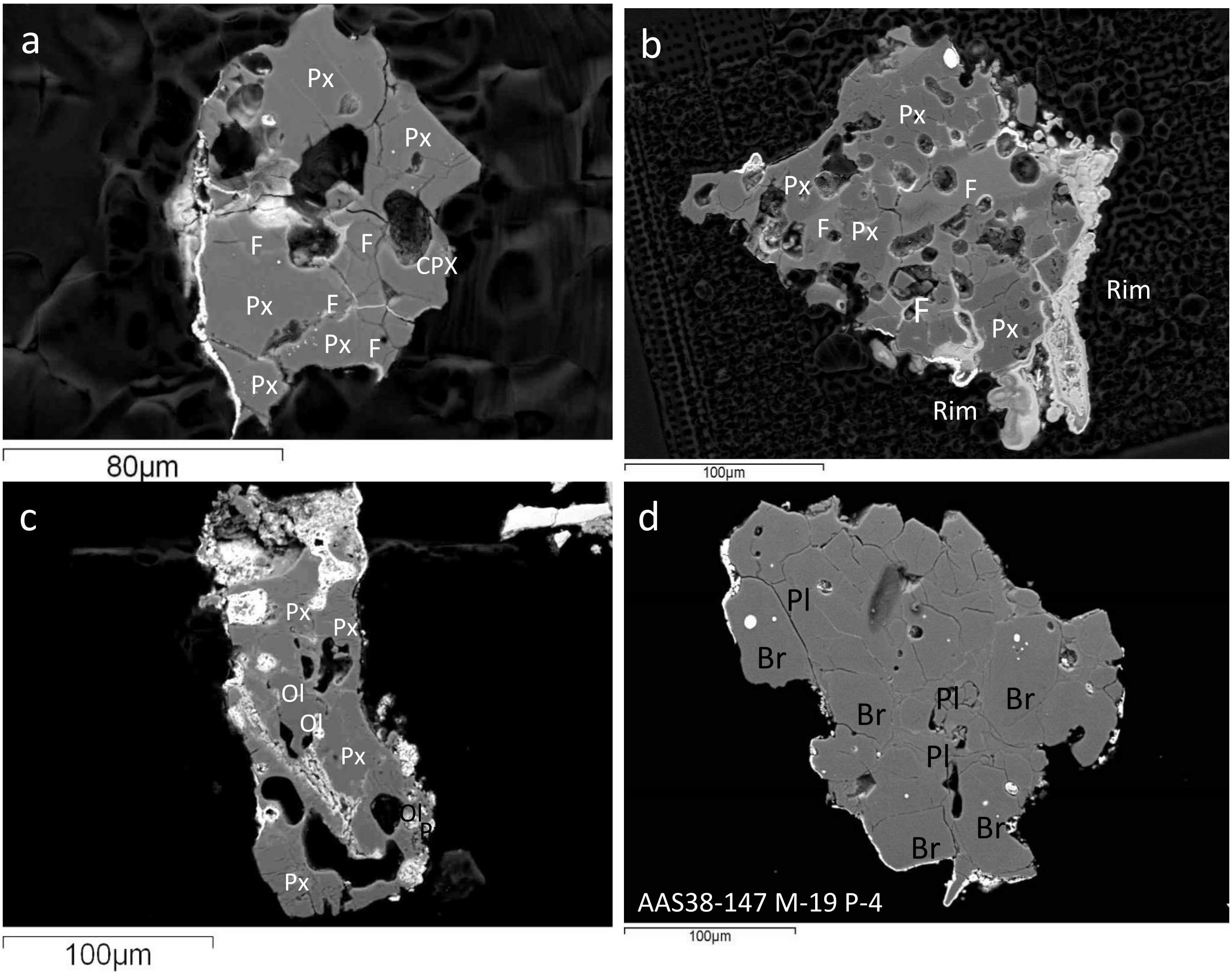

SUPPLEMENTARY FIGURE 3 : Chondrule Fragments

a. AAS38-147 M-14 P-2. IIB chondrule fragment with Ca-rich feldspar (An 93.7) labelled (F) and pyroxene (Fs-19.56,Wo-3.29) labelled (PX, CPX). A prominent partial rim can be seen

b. AAS38-153-M-8 P-2. type IB (CV chondritic) chondrule fragment comprising of Ca-rich feldspar (F) and enstatite (PX). A prominent partial rim is seen on the right side of the image.

c. **AAS38-147 M-5 P-15.** Type IIB chondrule (CV chondrite), olivine (Ol) (Fa19.9%) <10%, pyroxene (Fs-6.37,Wo-1.94) 80% & FeNiS metal 10%. A portion of the meteorite matrix is visible at the top. A partial rim at the right side top of the image.

d. **AAS38-147 M-19 P-4** :Type IIB Chondrite (L chondrite) comprising of hypersthene (Br) with Anorthite (Pl) and reduced metal and a prominent partial rim. FeNi metal (kamacite) blobs seen in hypersthene grains.

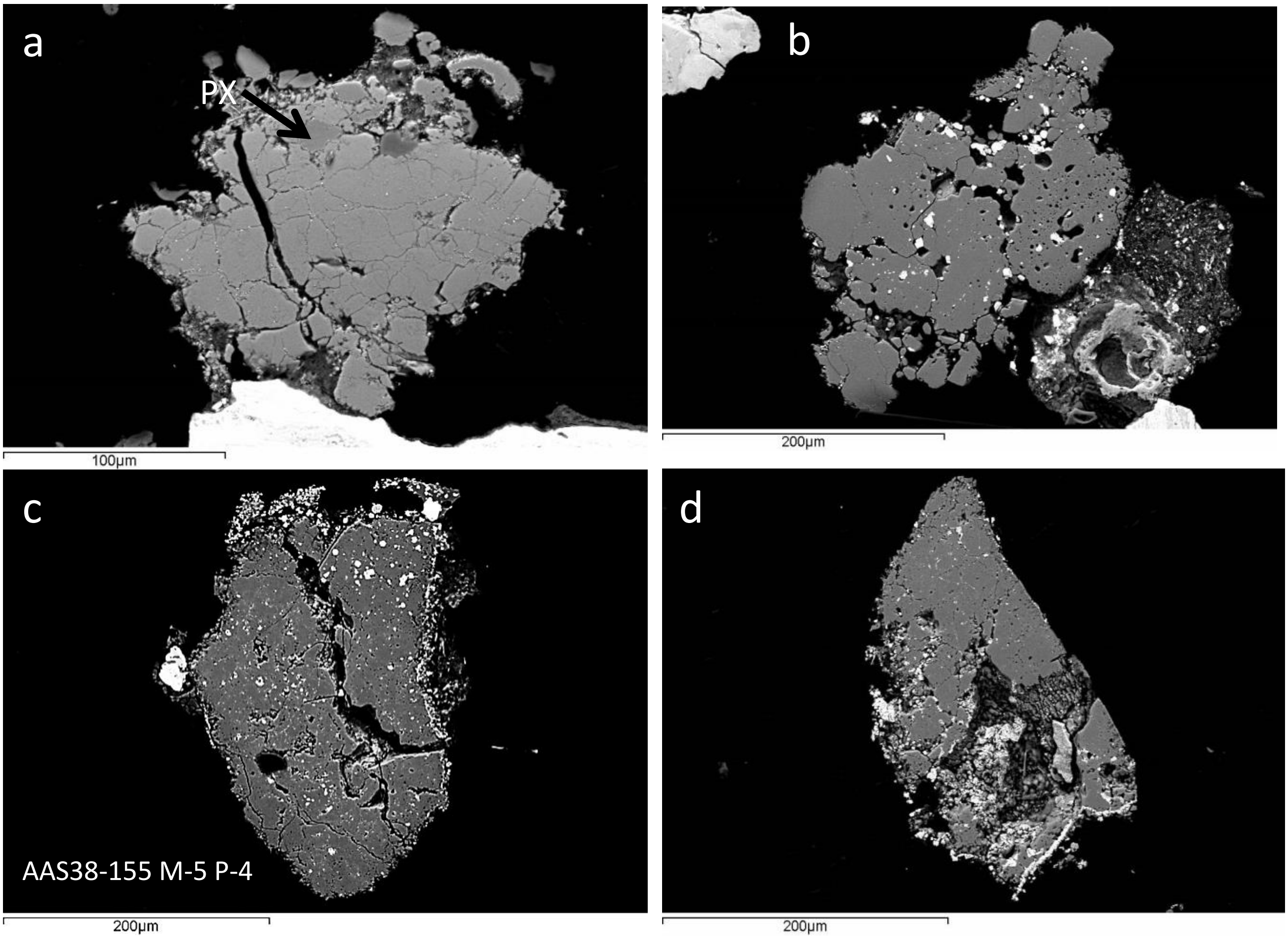

Supplementary Figure 4 : Hard, glassy matrices

a. **AAS38-165 M-10 P-2.** Olivine normative matrix with dessication cracks, and enstatite inclusions (PX).

b. **AAS38-153 M-22 P-3.** Fe-rich olivine with oxidized metal, matrix of an UOC.

c. **AAS38-155 M-2 P-1.** glassy, hardened matrix with dessication cracks.

d. **AAS38-155 M5 P-4.** Olivine normative matrix with dessication cracks and a partial rim (at the bottom of the picture).

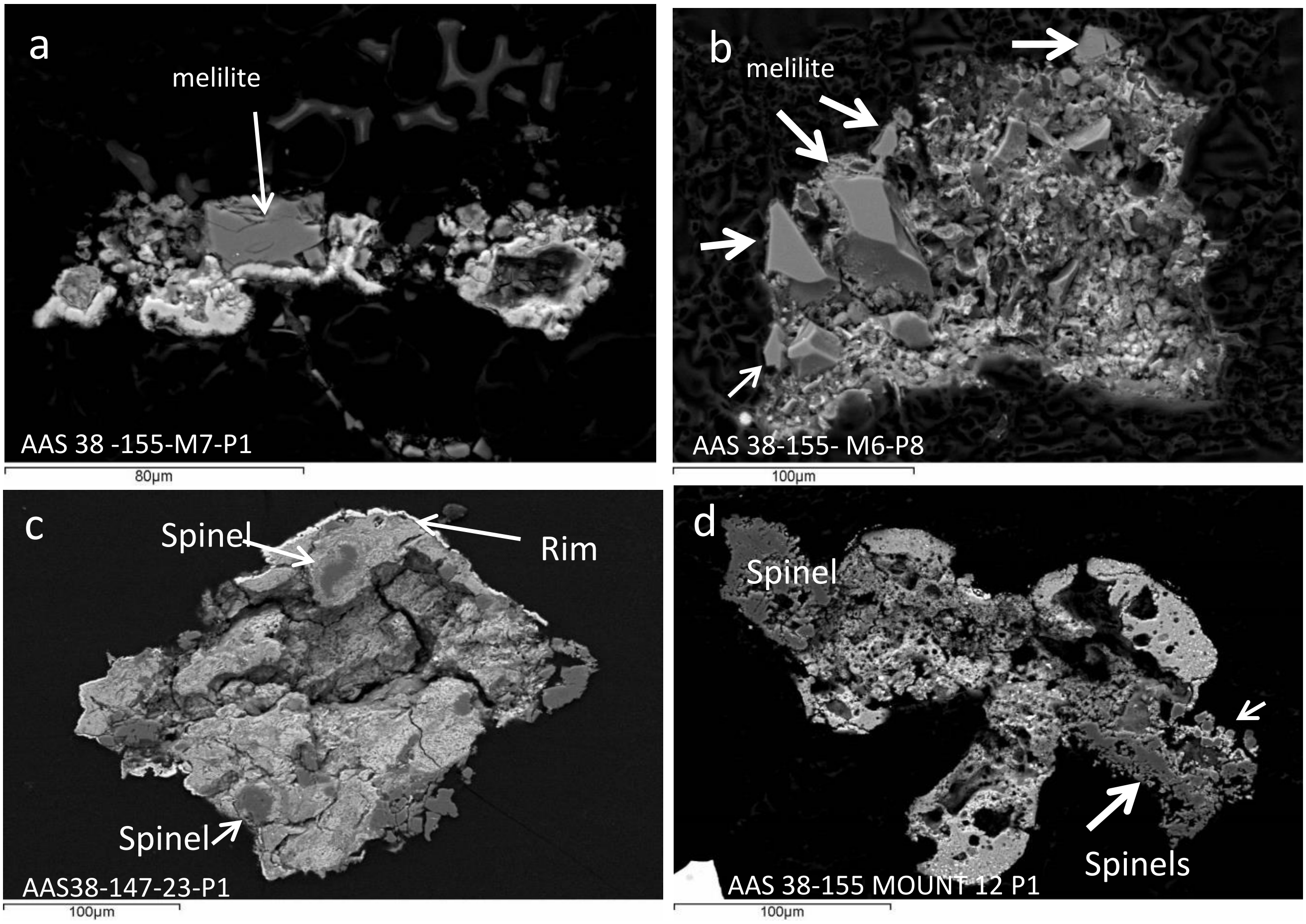

SUPPLEMENTARY FIGURE 5: Refractory phases

a. Melilite grain along with a thick rim of a particle. An empty space where another grain was dislodged is also visible.

b. Melilite grains showing similarities to those in CV chondrites

c. Spinel grains in a large umm with a partial rim.

d. Spinels enclosed in a scoriaceous matrix of a CV chondrite

SUPPLEMENTARY FIGURE 6.

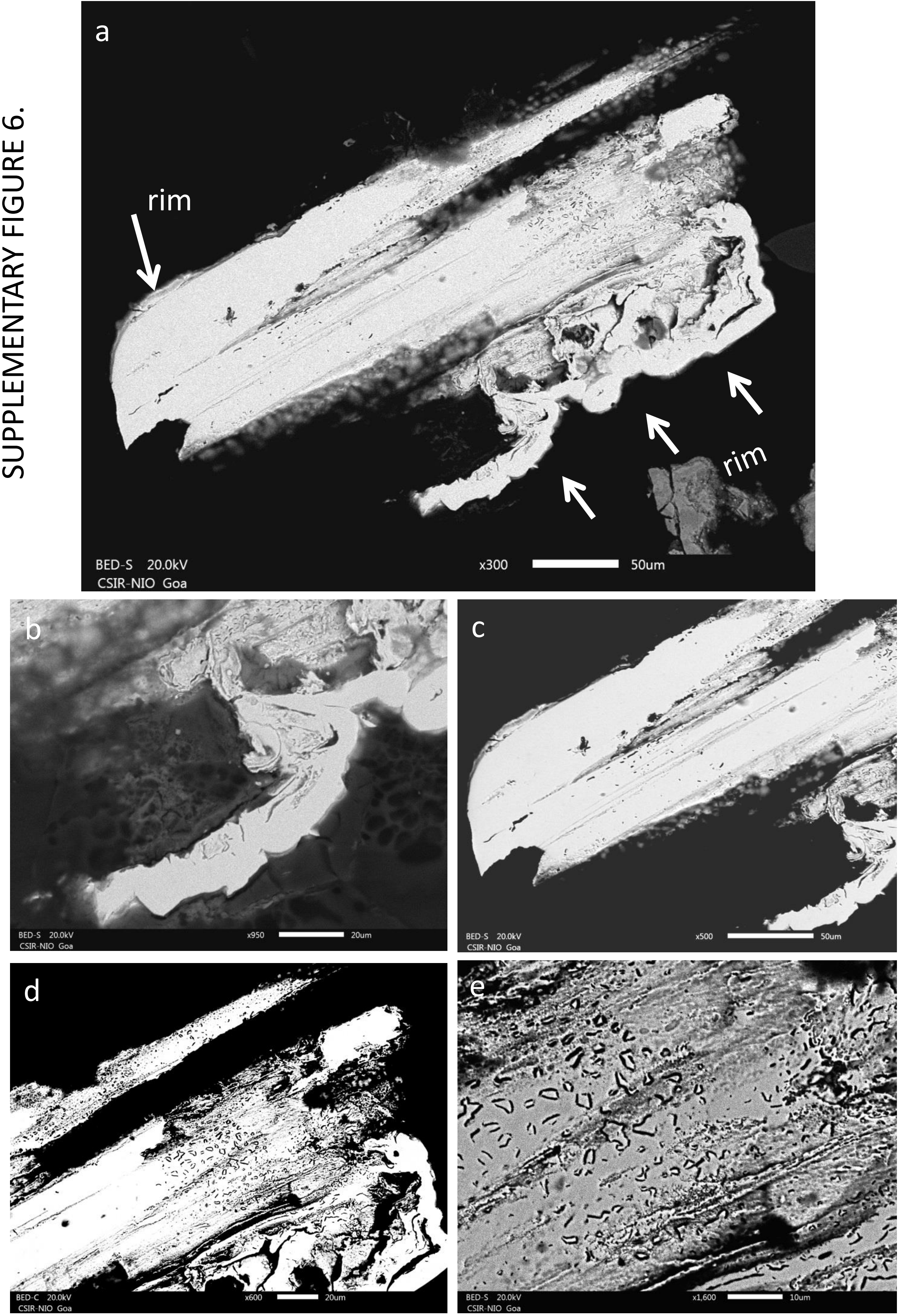

SUPPLEMENTARY FIGURE 6 : Native nickel particle (AAS38-165 M-4 P-1).

a. Full view showing a prominent rim in the bottom portion of the image and a small rim at the top portion.

b & c. Rim portion under higher magnification

d. a portion of the particle shows etching while on the seafloor, which is magnified in (**e**).

e. This particle apparently has a long residence time on the seafloor therefore it shows effects of etching , because nickel takes long residence time to undergo alteration.

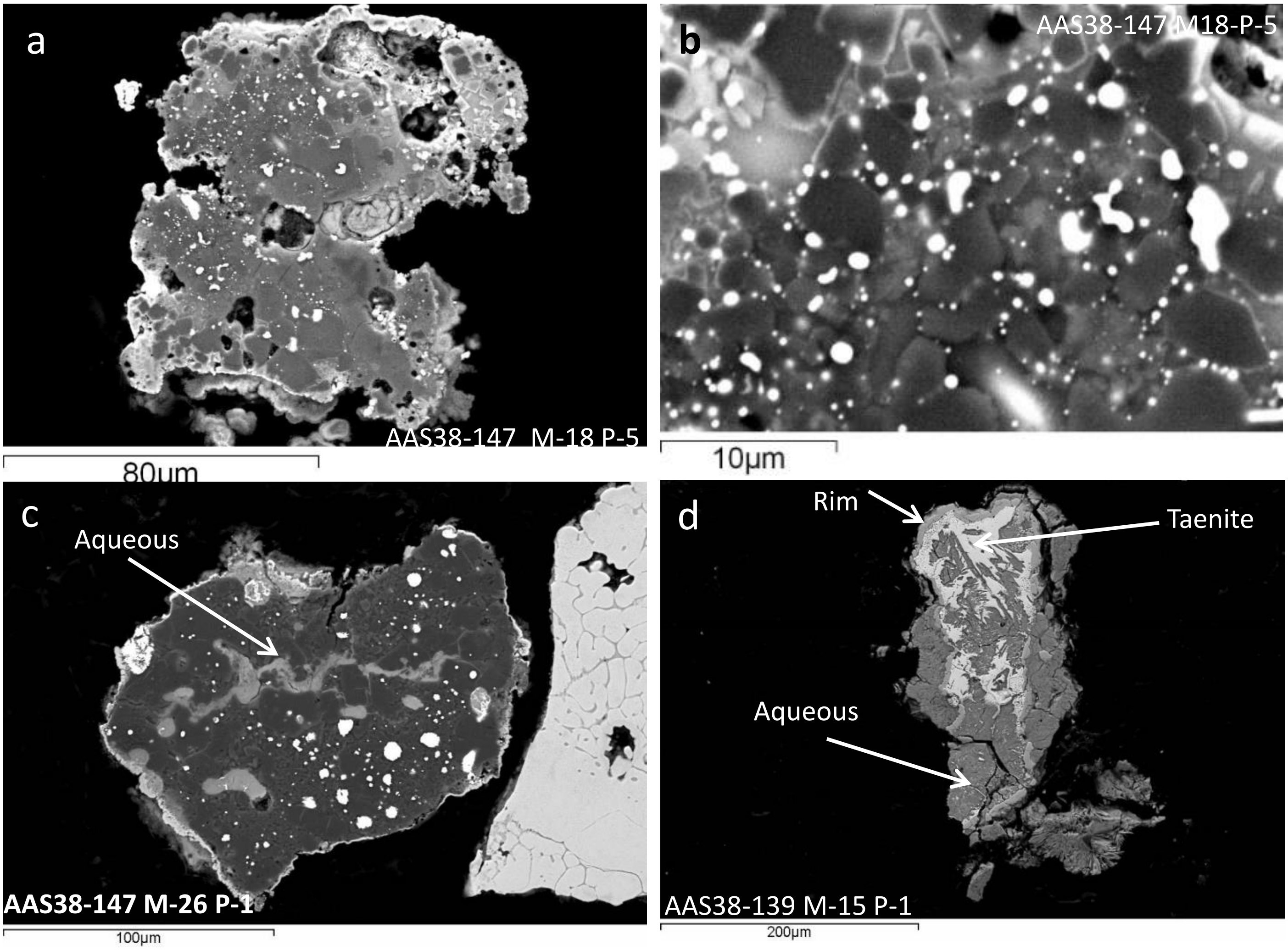

SUPPLEMENTARY FIGURE 7 : Metal-rich chondritic particles.

a. Metal-rich chondritic particle containing numerous blobs of kamacite.

b. Magnified portion of (**a**) showing a cluster of kamacite blobs.

c. In addition to kamacitic metal (shiny blobs), this particle also shows aqueous alteration which appears to be primary rather than that which took place on the seafloor as the alteration is in the central portions of the particle, seafloor aqueous alteration begins from all sides of the particles from and progressive inside the particle.

d. Taenite crystal with a rim. The particle has undergone aqueous alteration while on the seafloor, this phenomenon has been described in detail by Prasad et al. [26].
